# Patient-derived organoids as predictor of clinical response in ovarian cancer: a systematic review

**DOI:** 10.1016/j.tranon.2026.103017

**Published:** 2026-09-06

**Authors:** Merel Vegter, Prakriti Garkhail, Gatske M. Nieuwenhuyzen-de Boer, Ingrid A. Boere, Heleen J. van Beekhuizen

**Affiliations:** a Department of Gynecologic Oncology, Erasmus MC Cancer Institute, University Medical Center Rotterdam, the Netherlands; b Department of Obstetrics and Gynecology, Albert Schweitzer Hospital, Dordrecht, the Netherlands; c Department of Medical Oncology, Erasmus MC Cancer Institute, University Medical Center Rotterdam, the Netherlands

**Keywords:** Ovarian neoplasms, Organoids, Antineoplastic agents, Treatment outcome and neoplasm models, Experimental

## Abstract

Patient-derived organoids (PDOs) have emerged as promising preclinical models for functional drug testing in ovarian cancer, with potential to support personalized treatment selection. However, their predictive value for clinical treatment response remains unclear. This systematic review evaluated the predictive accuracy of ovarian cancer PDOs for treatment efficacy.

A systematic search of was conducted from inception to January 29, 2026. Eligible studies included patients with high-grade epithelial ovarian, fallopian tube, or primary peritoneal cancer from whom PDOs were generated for in vitro drug testing and directly correlated with clinical outcomes. The primary outcome was predictive accuracy, defined as concordance/ correlation between in vitro PDO drug response and in vivo patient outcomes. Because of substantial heterogeneity, a narrative synthesis was performed.

Twelve studies published between 2019 and 2026 were included. Cohort sizes ranged from 6 to 61 patients and comprised two prospective validation studies and ten retrospective translational or feasibility studies. Clinical endpoints were heterogeneous and included radiologic, biochemical and histopathologic response, progression-free survival, and descriptive clinical course. Four studies reported statistically significant associations between PDO drug response and clinical outcome, six reported concordant findings without formal statistical testing, two showed limited or mixed concordance, and one relied on descriptive comparison only. The strongest evidence came from two prospective studies, which reported accuracies of 89% and 91.7%.

Ovarian cancer PDOs show promise as functional predictors of treatment response, but current evidence is limited by small cohorts, methodological heterogeneity, and scarce prospective validation. Standardized prospective studies are needed to define their clinical applicability.

## Highlights

• Systematic review of 12 studies linking ovarian cancer PDOs to clinical outcomes.
• Prospective studies reported PDO predictive accuracies of 89% and 91.7%.
• Evidence supports PDOs as promising functional predictors of treatment response.

## Graphical abstract

**Figure:**
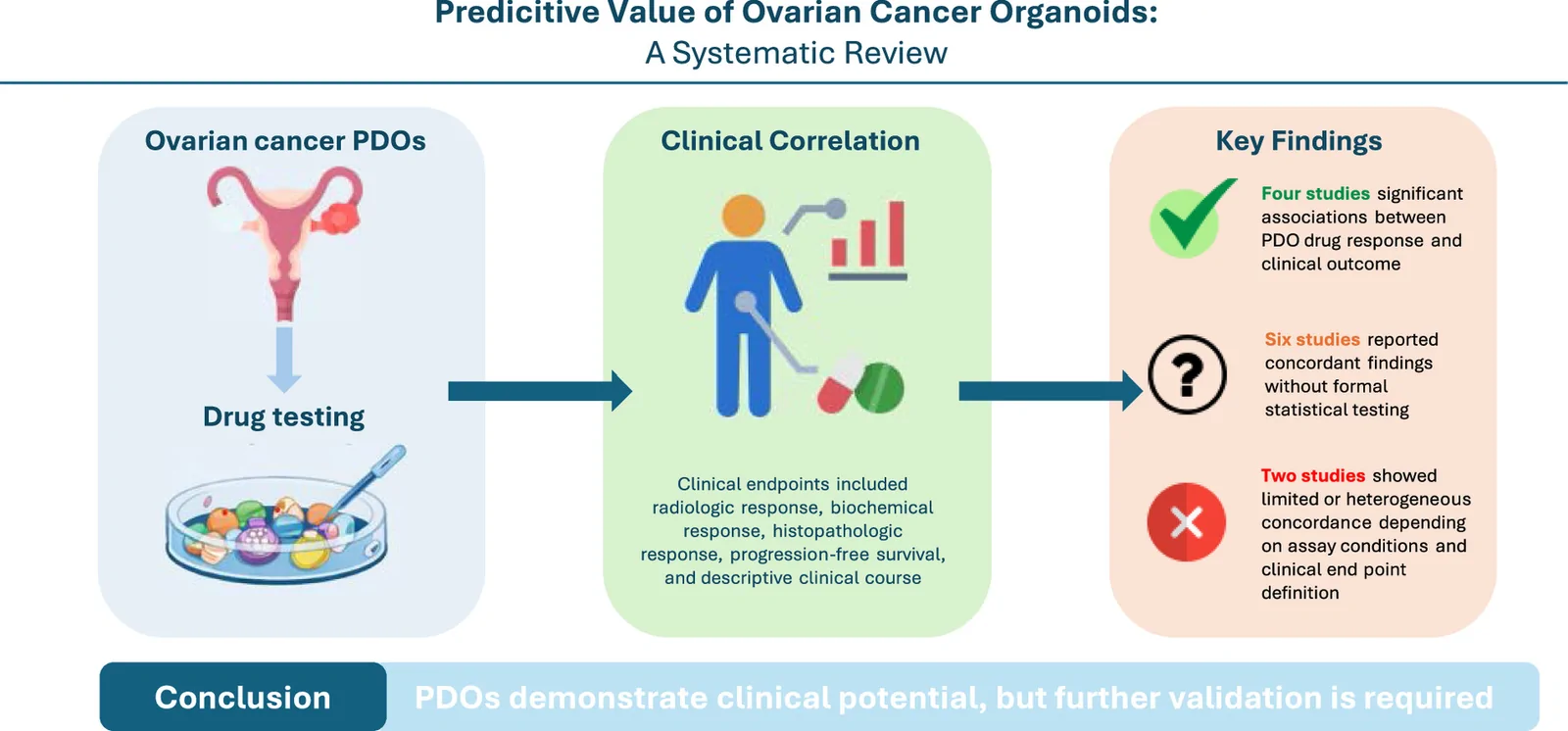

## Introduction

Ovarian cancer is one of the most lethal gynecologic malignancies, accounting for over 300,000 new cases and nearly 200,000 deaths annually [1], and is characterized by considerable interpatient variability in treatment response despite largely standardized treatment approaches. Most patients are diagnosed at advanced stages, when five-year survival rates drop below 30% despite intensive multimodal treatment [2].

Although standard therapy, including cytoreductive surgery, platinum-based chemotherapy, and targeted agents such as angiogenesis inhibitors and poly (ADP-ribose) polymerase inhibitors (PARPi), can induce initial responses, relapse is common due to intrinsic or acquired drug resistance [3]. Relapsed disease is often treated with additional platinum- or non-platinum-based chemotherapy, and emerging therapeutic strategies, including antibody–drug conjugates (ADCs) and immunotherapies such as PD-1 inhibitors and bispecific antibodies, are increasingly being evaluated in clinical practice and clinical trials [4,5]. As the number of available treatment options continues to expand, selecting the most effective therapy for each individual patient remains a major challenge. The inability to accurately predict therapeutic benefit therefore continues to hinder personalized treatment and improvement of patient outcomes.

Conventional preclinical models, including two-dimensional (2D) cell lines and patient-derived xenografts (PDX), have been essential for understanding ovarian cancer biology and testing new therapeutics. However, their translational utility is limited. Cell lines often fail to preserve the genetic diversity and tumor microenvironment of primary disease, while PDX models, though more representative, are time-consuming, expensive, and not scalable to the clinical timescale needed for patient care [[6], [7], [8]]. Consequently, neither model is optimal for real-time prediction of individual patient responses.

Over the past decade, three-dimensional Patient-Derived Organoid (PDO) models have emerged as a promising alternative. Organoids are miniaturized tumor cultures established directly from patient-derived tissue, grown in extracellular matrix scaffolds that allow them to maintain architectural, genomic, and phenotypic features of the original tumor [9]. Importantly, PDOs can be expanded and tested against multiple drugs within weeks, making them potentially suitable for informed clinical decision-making. Despite these advantages, conventional PDO models primarily consist of epithelial tumor cells and do not recapitulate the native tumor microenvironment. Consequently, interactions with immune cells, cancer-associated fibroblasts, vascular components, and extracellular matrix are only partially represented, which may limit their ability to predict responses to immunotherapies and other microenvironment-targeting treatments.

Early reports in ovarian cancer suggest that PDOs retain intra- and interpatient heterogeneity in drug response, and that PDO chemosensitivity profiles have been reported to correlate with clinical outcomes [10,11]. For example, carboplatin resistance observed in ovarian cancer organoids has been shown to mirror clinical resistance patterns [12], while individual case reports suggest PDO-guided testing may reveal unexpected therapeutic opportunities [13].

However, evidence remains fragmented due to heterogeneous methodologies for tissue sampling, organoid generation, and drug-response assessment, as well as technical challenges that affect reproducibility and organoid establishment. Consequently, reported concordance between PDO responses and clinical outcomes varies, while most studies are limited by small sample sizes and a lack of prospective validation. These limitations raise important questions regarding the predictive accuracy and clinical applicability of ovarian cancer PDOs, warranting a systematic review of the available evidence.

This systematic review aims to consolidate and critically appraise the existing evidence on the predictive value of ovarian cancer PDOs. The primary outcome of interest is the predictive accuracy of PDOs for treatment efficacy, defined as the correlation or concordance between in vitro organoid drug responses and in vivo clinical patient outcomes. By pooling data across studies, this review aims to quantify the predictive performance of PDOs, assess methodological strengths and limitations, and identify critical knowledge gaps for future research.

## Methods

### Protocol and registration

This systematic review was conducted in accordance with the Preferred Reporting Items for Systematic Reviews and Meta-Analyses (PRISMA) 2020 guidelines [14]. The protocol was prospectively registered in PROSPERO (CRD42025635321: Predictive Power of Ovarian Cancer Organoids for Targeted Therapy Efficacy In Vivo). The primary outcome of interest was the predictive accuracy of PDOs for treatment efficacy, defined as the correlation or concordance between in vitro and in vivo results.

### Eligibility criteria

Inclusion criteria were studies involving patients with high-grade epithelial ovarian cancer, including fallopian tube and primary peritoneal cancer, in the primary, metastatic, or recurrent setting, from whom tumor tissue was used to generate patient-derived organoids. Eligible studies used ovarian cancer PDOs for in vitro drug testing and directly correlated these responses with clinical outcomes. Clinical outcomes serving as comparators included radiological or clinical response (according to Response Evaluation Criteria in Solid Tumors [RECIST] 1.1 [22]), biochemical response (e.g., CA-125), histopathologic response (using the Chemotherapy Response Score (CRS) system (CRS1 = minimal/no response, CRS2 = appreciable response, CRS3 = complete/near-complete response)), progression-free survival (PFS), or descriptive clinical courses (e.g., whether the patient had no evidence of disease during follow-up). Eligible study designs comprised experimental preclinical–clinical correlation studies and both retrospective and prospective cohort studies linking organoid drug responses to patient outcomes.

Exclusion criteria were studies unrelated to ovarian cancer, studies using non-human or animal-derived organoids, engineered cell lines, or models not derived from patient tumor tissue, and studies focusing solely on technical aspects of organoid culture without therapeutic evaluation. Conference abstracts published before 2021, registry records without data, non-English publications, reviews, single case reports, editorials and non-peer reviewed manuscripts were not considered.

### Information sources and search strategy

A comprehensive search was developed in collaboration with an experienced medical information specialist. The following databases were searched from inception to January 29, 2026: MEDLINE ALL (Ovid, 1946–present), Embase (Embase.com, 1971–present), Web of Science Core Collection (Clarivate, 1975–present), Cochrane Central Register of Controlled Trials (Wiley, 1992–present) and Google Scholar (via Publish or Perish, top 50 results screened).

Search strategies combined controlled vocabulary and free-text terms for “ovarian cancer,” “organoids,” and “treatment response.” Full strategies for each database are provided in Supplementary Table S1.

### Study selection

All records were imported into Covidence, and duplicates were removed. Two reviewers (MV, PG) independently screened titles and abstracts for eligibility, followed by full-text review of potentially relevant articles. Discrepancies were resolved by discussion. The selection process will be reported in a PRISMA flow diagram.

### Data extraction

Extracted variables included: study details (authors, year, design), patient and sample characteristics, organoid derivation and culture methods, drugs tested (chemotherapy or targeted therapies), in vitro drug sensitivity assays, clinical outcomes, and concordance/correlation metrics between PDOs and patient responses.

### Risk of bias assessment and data synthesis

No standardized or validated risk-of-bias assessment tool currently exists for laboratory-based or in vitro studies, and established tools developed for clinical trials or observational research are not directly applicable to preclinical experimental platforms. Therefore, a formal risk-of-bias assessment using a standardized scoring system was not performed. Instead, a descriptive, qualitative domain-based approach was used to identify potential sources of bias relevant to organoid-based drug testing studies. The following domains were considered: (i) patient selection and tissue availability; (ii) organoid derivation success and attrition; (iii) fidelity of organoids to the original tumor (e.g., histologic or molecular validation); (iv) drug exposure design and response metrics; (v) definition and determination of clinical outcomes; and (vi) study design (prospective vs retrospective). These methodological considerations were described across the tables and used to inform interpretation of the results but were not applied as exclusion criteria.

Given the substantial heterogeneity in experimental platforms, drug testing methodologies, clinical endpoints, and outcome definitions, quantitative meta-analysis was not performed. Instead, a narrative, theme-based synthesis was undertaken, in which data were systematically compared and qualitatively integrated across studies. This approach allowed identification of consistent patterns, similarities, and differences in reported outcomes, as well as evaluation of concordance between organoid drug responses and clinical treatment outcomes.

For data synthesis, studies were grouped according to predefined methodological domains, including organoid derivation and culture strategies, drug sensitivity assessment, clinical endpoint definitions, and reported concordance between organoid response and patient outcomes. When studies reported multiple clinical endpoints for treatment response, the endpoint designated by the authors as the primary or most clinically relevant outcome was used for synthesis.

## Results

### Study selection

**Fig. 1:**
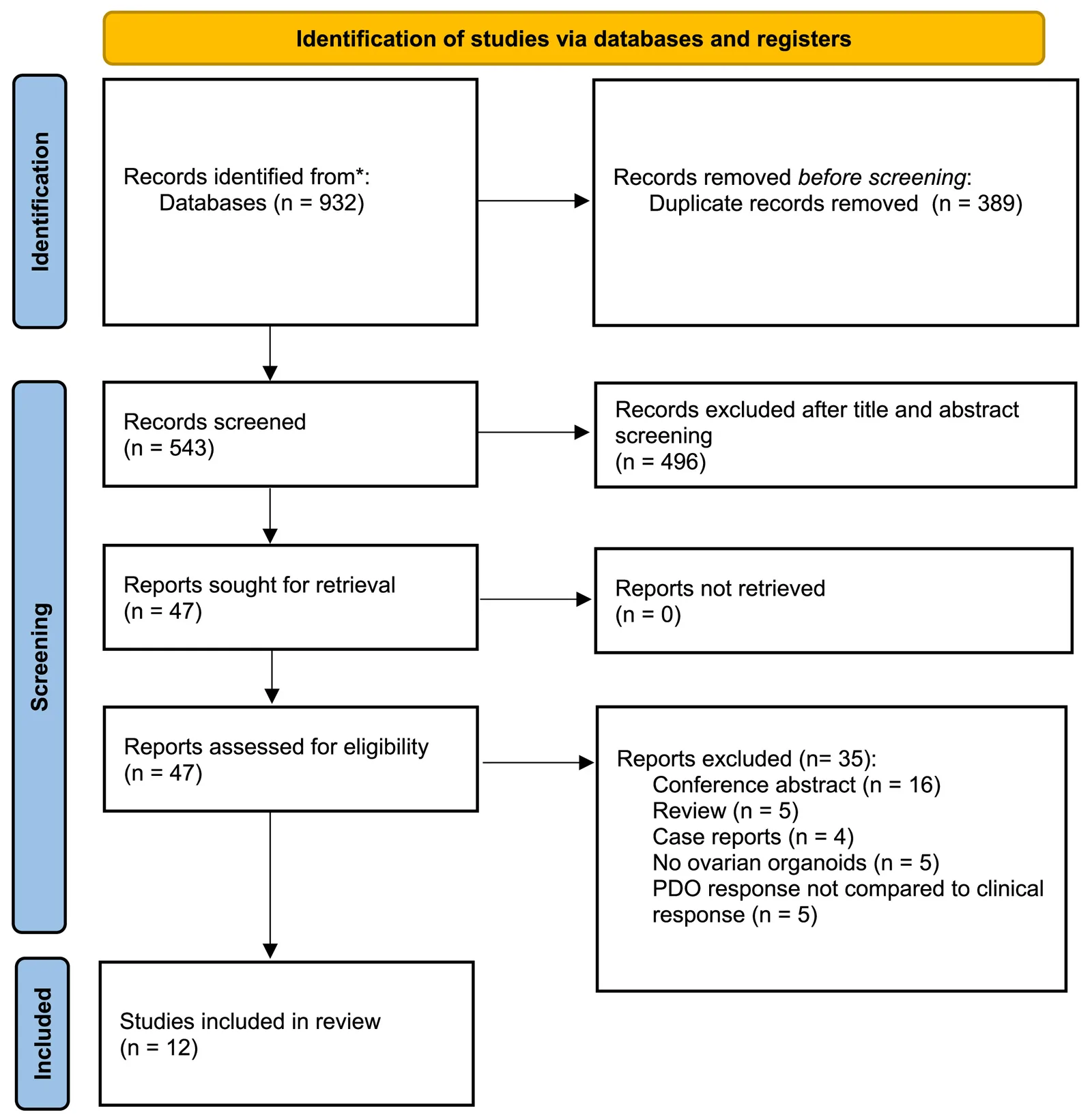
PRISMA 2020 flow diagram of study identification, screening, eligibility assessment, and inclusion. A systematic literature search was performed from database inception to January 29, 2026, using MEDLINE ALL (Ovid), Embase (Embase.com), Web of Science Core Collection, the Cochrane Central Register of Controlled Trials, and Google Scholar. After duplicate removal, records were screened by title and abstract, followed by full-text assessment for eligibility. Full-text articles were excluded for the following reasons: conference abstracts (n = 16), reviews (n = 5), case reports (n = 4), did not include ovarian cancer organoids (n = 5), or did not correlate patient-derived organoid responses with clinical treatment response (n = 5). Twelve studies met the eligibility criteria and were included in the qualitative synthesis.

A total of 932 records were identified through bibliographic database searching and Google Scholar (MEDLINE, n = 187; Embase, n = 471; Web of Science, n = 224; Cochrane, n = 0; Google Scholar, n = 50) (Table 1). After deduplication within each source, 543 unique records remained and were screened based on title and abstract. Of these, 47 reports were retrieved for full-text eligibility assessment. Following full-text screening, twelve studies met the inclusion criteria by linking drug response in patient-derived ovarian cancer organoids to a corresponding clinical treatment response and were included in this systematic review (Fig. 1).

**Table 1:** Identified records after database searching.

Database	Source / Coverage period	Records identified	Records after duplicates removed
MEDLINE ALL	Ovid, 1946–present	187	183
Embase	Embase.com, 1971–present	471	296
Web of Science Core Collection	Clarivate, 1975–present	224	52
Cochrane Central Register of Controlled Trials	Wiley, 1992–present	0	0
Google Scholar	via Publish or Perish, top 50 results screened	50	12
Total		932	543

### Study characteristics

The included studies were published between 2019 and 2026. Cohort sizes ranged from 6 [15] to 61 patients [16]. Study designs included two prospective validation studies [17,18], one of which was blinded, and multiple retrospective translational or feasibility studies [[10], [11], [12],^15^,^16^,[19], [20], [21], [22], [23]]. The studies used diverse approaches to PDO derivation, culture, and drug sensitivity testing. Key study characteristics are summarized in Table 2, while detailed information on organoid derivation, culture conditions, validation, and drug testing methodologies is provided in Table S2. Across studies, clinical endpoints used to classify treatment response were heterogeneous and included radiological response rate according to RECIST 1.1 [24], biochemical response by measuring blood CA-125 decline or normalization (typically defined as ≤35 U/mL, based on GCIG criteria [25]), histopathologic response scores (chemotherapy response score [CRS]), PFS and descriptive clinical courses.

**Table 2:** Characteristics of organoid derivation and drug testing.

Study	Year	Study design	N patients / N models	Ovarian cancer subtype(s)	3D model type	Tumor tissue source	Drug classes tested	Clinical endpoint(s) used
Shuford et al. [17]	2019	Prospective, blinded validation	44 / 44	HGSC	Short-term ex vivo 3D spheroids	Primary tumor biopsies	Platinum, taxane, others	CA-125, radiologic response (RECIST based), PFS
de Witte et al. [11]	2020	Retrospective translational correlation	23 / 36	HGSC	Long-term PDOs	Tumor tissue, ascites	Platinum, taxane, targeted agents. others	Chemotherapy Response Score (CRS), CA-125, radiologic response (RECIST based), PFS
Gorski et al. [12]	2021	Retrospective cohort	6 / 6	HGSC	Long-term PDOs	Surgical tumor tissue	Platinum	PFS
Tao et al. [19]	2022	Retrospective translational correlation	7 / 7	HGSC, EC, CCC	Long-term PDOs	Tumor tissue	Platinum, PARPi	CA-125, radiologic response (RECIST based), recurrence
Jun et al. [20]	2023	Feasibility (with clinical linkage)	10 / 10	HGSC, LGSC, CCC, EC, carcinosarcoma	Organo-on-pillar PDOs (short-term)	Surgical tumor tissue	Platinum	CA-125, clinical course
Senkowski et al. [21]	2023	Platform + retrospective correlation	10 / 17	HGSC	Long-term PDOs	Tumor tissue, ascites	Platinum, taxane, targeted agents, other	CA-125
Ito et al. [16]	2023	Retrospective cohort	61 / 61	Serous, CCC, EC, mucinous	Cancer tissue-originated spheroids	Tumor tissue, ascites	Platinum, taxane	Radiologic response (RECIST based), CA-125
Chang et al. [15]	2024	Feasibility/ biobank, clinical correlation	13 / 7	HGSC, CCC, mucinous, carcinosarcoma	Long-term PDOs	Tumor tissue (incl. metastasis)	Platinum, taxane, others	Clinical course
Chen et al. [10]	2024	Feasibility + case-level clinical correlation	38 / 31	HGSC, CCC, mucinous, EC	Long-term PDOs	Surgical tumor tissue	Platinum, taxane, others	Platinum sensitivity category
Nikeghbal et al. [22]	2025	Retrospective feasibility/translational- correlation	5/5	HGSOC	Long-term PDOs	Ascites	Platinum, taxane	Platinum sensitivity category
Thorel et al. [23]	2025	Retrospective translational correlation	31/37	HGSOC, OCCC, mucinous, carcinosarcoma, EC	Long-term PDOs	Tumor tissue, ascites	Platinum, taxane, PARPi	PFS, PFI, OS, CA-125, KELIM
Wang et al. [18]	2026	Prospective correlation	30/21	HGSOC, OCCC, EC	Long-term PDOs	Tumor tissue, ascites	Platinum, taxane, PARPi	Radiologic response (RECIST based), PFI, PFS, PARPi maintenance response

Abbreviations: CA-125: Cancer Antigen 125; CCC: Clear Cell Carcinoma; CRS: Chemotherapy Response Score; EC: Endometrioid Carcinoma; EOC: Epithelial Ovarian Cancer; FT: Fallopian Tube; HGSC: High-Grade Serous Carcinoma; HGSOC: High-Grade Serous Ovarian Cancer; KELIM: CA-125 Elimination Rate Constant K; LGSC: Low-Grade Serous Carcinoma; OCCC: Ovarian Clear Cell Carcinoma; OS: Overall Survival; PARPi: Poly(ADP-ribose) Polymerase Inhibitor; PDO: Patient-Derived Organoid; PFI: Platinum-Free Interval; PFS: Progression-Free Survival; PP: Primary Peritoneal; RECIST: Response Evaluation Criteria in Solid Tumors.

### Organoid derivation, culture, tumor–PDO comparability and drug testing

Across the included studies, PDOs and related 3D tumor models were established from a range of ovarian cancer tissue sources, including primary ovarian tumors, metastatic lesions, and malignant ascites. Most studies focused on epithelial ovarian cancer, with high-grade serous carcinoma as the predominant subtype [11,12,21,22], while clear cell, endometrioid, mucinous, low-grade serous, and carcinosarcoma subtypes were also represented in several cohorts [10,[15], [16], [17], [18], [19], [20],^23^].

#### Organoid derivation and establishment success

Organoid derivation was performed using freshly obtained surgical specimens or ascites-derived tumor cells, which were mechanically and/or enzymatically dissociated prior to three-dimensional culture. Establishment success rates varied substantially across studies and experimental platforms. When reported, successful PDO generation varied widely across studies, ranging from approximately 50–70% in long-term expandable PDO platforms [18,21] to over 80% in short-term models [16], depending on tissue source, culture approach, and the definition of successful establishment. Several studies reported discrepancies between the number of patients included and the number of PDO lines successfully established, reflecting multiple derivation attempts per patient or failure of organoid outgrowth from individual specimens [10,15,23]. Detailed establishment rates and denominators for each study are provided in Table S2.

Overall, variability in PDO establishment success was associated with differences in tissue source, dissociation protocols, extracellular matrices, culture media, and study-specific definitions of successful establishment. Direct comparisons between these variables were not possible due to inconsistent reporting across studies. Similarly, although several studies included patients who had received prior chemotherapy, its influence on establishment success could not be systematically assessed.

#### Culture strategies: short-term versus long-term models

Following derivation, studies employed either short-term culture systems [16,17,20] or long-term expandable PDO platforms [[10], [11], [12],^15^,^18^,^19^,[21], [22], [23]]. Short-term models were typically established and tested within days, did not undergo sustained expansion or biobanking, and involved limited or no passaging, thereby prioritizing rapid turnaround and clinical feasibility [16,17,20]. In contrast, long-term PDO platforms allowed sustained expansion, cryopreservation, repeated drug testing, and longitudinal molecular and histopathologic characterization, but generally required longer establishment periods and were more commonly used in retrospective or translational correlation studies [[10], [11], [12],^15^,^18^,^19^,[21], [22], [23]].

#### Tumor vs PDO comparability (fidelity to the original tumor)

Assessment of tumor vs PDO comparability was performed in 10 of the 12 included studies (83%) [[10], [11], [12],^15^,^16^,[18], [19], [20], [21],^23^]. When evaluated, fidelity to the original tumor was examined using histopathologic comparison, immunohistochemistry, genomic profiling, transcriptomic analysis, or combinations thereof.

Histologic and immunohistochemical comparison was the most frequently applied approach to assess tumor–PDO comparability, particularly in studies using short-term based culture systems. These analyses demonstrated preservation of key morphologic characteristics and tumor marker expression consistent with the original tumors. Reported markers included subtype-specific and ovarian cancer–associated proteins such as p53, estrogen receptor, HNF1β, Claudin-4, PAX8, and WT1, as well as maintenance of mucin production in organoids derived from mucinous carcinomas [16,20]. Together, these findings support preservation of tumor identity in short-term organoid platforms.

Several studies using long-term expandable PDO platforms performed more extensive molecular validation. These included paired genomic and transcriptomic analyses demonstrating retention of key driver alterations and overall molecular profiles relative to the original tumor tissue, as well as assessment of genetic or phenotypic stability across passages [11,12,15,18,19,21,23]. Together, these analyses indicated that PDOs retained key biological characteristics of the original tumors.

Two studies (17%) did not perform explicit paired histologic or molecular comparison between the tumor and derived 3D model and relied primarily on functional drug response as evidence of model relevance [17,22]. A detailed overview of tumor–PDO comparability assessments performed in each study is provided in Table S2.

#### Drug testing and in vitro response readouts

Drug sensitivity testing approaches varied substantially between studies. Most studies evaluated standard-of-care agents, particularly platinum compounds and taxanes, while several also included targeted therapies such as PARPi or anti-angiogenic agents. Drug testing was conducted using either single-agent exposures or, in some studies, combination regimens intended to reflect clinical treatment protocols. Readout timing ranged from short-term viability assessment within 3-7 days to longer exposure periods extending beyond one to two weeks in long-term expandable PDO platforms [11,12,15,18,19,[21], [22], [23]].

Drug response was quantified using a variety of assays, including luminescence-based ATP viability measurements, metabolic or imaging-based readouts, and relative growth inhibition–based response metrics. Response classification strategies included IC50 or EC50 values, area-under-the-curve analyses, and binary sensitive or resistant thresholds defined relative to clinically achievable drug concentrations. This methodological heterogeneity in drug exposure, readout timing, and response definition is summarized in Table S2. As a result, direct quantitative comparison of PDO performance across studies was not feasible, supporting the use of a narrative synthesis approach in this review.

### Concordance between clinical and PDO response

Across the included studies, clinical treatment response was defined using heterogeneous endpoints reflecting differences in study design and treatment context. Reported endpoints included radiologic response assessed by RECIST1.1, biochemical response based on CA-125 decline or normalization, histopathologic response scores following neoadjuvant therapy, PFS and descriptive clinical courses (e.g., no evidence of disease status, survival, follow-up duration, and CA-125 trends without formal response criteria) [15]. Several studies reported more than one clinical endpoint [10,11,[15], [16], [17], [18], [19], [20],^22^], whereas others relied on a single primary outcome [12,21,23]. An overview of clinical endpoints and concordance metrics per study is provided in Table 3, with detailed study-level definitions and analyses summarized in Table S3.

**Table 3:** Concordance between organoid drug response and patient clinical outcomes.

Study	Clinical therapy	In vitro drug testing	Endpoint’s	Concordance between PDO and clinical outcome
Shuford et al. [17]	Platinum-taxane combination therapy	Single-agent panel	CA-125 → imaging → PFS	Prospective validation: 89% accuracy, sensitivity 86%, specificity 100%; significant PFS stratification (short vs. long PFS).
de Witte et al. [11]	Platinum-taxane combination therapy ± targeted agents	Single agents and combinations	CRS → CA-125 → Radiologic response (RECIST) → survival	Significant separation of PDO responses between clinically sensitive vs resistant groups across all endpoints (p<0.01)
Gorski et al. [12]	Platinum-based chemotherapy	Carboplatin	PFS	PDO-defined carboplatin resistance associated with shorter PFS (p=0.025)
Tao et al. [19]	Platinum-taxane combination	Cisplatin	CA-125 → recurrence timing	PDO cisplatin response correlated with CA-125 normalization (p=0.0047); RECIST not discriminative
Jun et al. [20]	Platinum-taxane ± bevacizumab	Carboplatin	CA-125 / clinical course	Patient-linked proof-of-concept concordance; no cohort-level metric
Senkowski et al. [21]	Carboplatin + paclitaxel	Combination and single agents	CA-125	Significant correlation only when PDOs cultured in Human Plasma-Like Medium (HPLM) (r=0.77, p=0.013)
Ito et al. [16]	Carboplatin + paclitaxel	Carboplatin + paclitaxel	Radiologic response (RECIST) → CA-125	Combined in vitro sensitivity classification significantly associated with RECIST response (p=0.0037)
Chang et al. [15]	Platinum-based regimens ± bevacizumab	Single-agent panel	Clinical course	Case-level concordance reported; insufficient follow-up for accuracy metrics
Chen et al. [10]	Multiple regimens (platinum-sensitive to refractory)	Single-agent panel	Platinum sensitivity category	Case-based concordance: PDO resistance patterns aligned with refractory clinical courses
Nikeghbal et al. [22]	Carboplatin + paclitaxel	Carboplatin + paclitaxel	Platinum sensitivity category	Case-level concordance: PDO viability responses matched clinical platinum sensitivity classification (sensitive cases showed viability reduction to drugs; resistant/refractory showed carboplatin and combination resistance, with variable paclitaxel monotherapy response); no cohort-level accuracy/sensitivity metrics reported.
Thorel et al. [23]	Platinum-based regimens ± bevacizumab± targeted agents	Single agents and combinations	PFS, PFI, OS,CA-125, KELIM	Association with PFI: Carboplatin PDO response stratified patients into resistant vs sensitive groups with shorter vs longer PFI (median 1.61 vs 6.80 months; p=0.06); discrepant cases (carboplatin-sensitive PDO with clinically platinum-resistant relapse) reported; mixed concordance for PFS/OS/CA-125 and no correlation with KELIM.
Wang et al. [18]	Platinum-based regimens ± targeted agents	Single-agent panel	RECIST, PFI, PFS, PARPi maintenance response	Prospective validation: PDO responses predicted first-line chemo response with sensitivity 100%, specificity 66.7%, accuracy 91.7%, AUC 0.95, κ=0.75; functional PARPi response added predictive value beyond genomic HRD status.

Abbreviations: AUC: Area Under the Curve; CA-125: Cancer Antigen 125; CRS: Chemotherapy Response Score; HPLM: Human Plasma-Like Medium; HRD: Homologous Recombination Deficiency; KELIM: CA-125 Elimination Rate Constant K; OS: Overall Survival; PARPi: Poly(ADP-ribose) Polymerase Inhibitor; PDO: Patient-Derived Organoid; PFI: Platinum-Free Interval; PFS: Progression-Free Survival; RECIST: Response Evaluation Criteria in Solid Tumors.

Organoid-clinical concordance was evaluated across all twelve included studies, with variability in the methods used to quantify and report associations. Four studies demonstrated statistically significant associations between PDO drug response and clinical outcome [11,12,16,17]. Five studies reported concordant findings without formal statistical testing [10,18,20,22,23], while two studies showed limited or heterogeneous concordance depending on assay conditions and clinical end point definition [19,21]. One study relied solely on descriptive comparison of PDO drug response with the clinical disease course, without performing formal concordance analysis or statistical testing [15].

In a retrospective cohort with 23 evaluable patients, PDO drug sensitivity differed significantly between clinically defined responder and non-responder groups across multiple clinical endpoints, including radiologic response, CA-125 response, and histopathologic response scores, demonstrating concordance at the group level [11]. In a smaller cohort focused on platinum resistance (n = 6), PDO-defined carboplatin resistance was significantly associated with shorter PFS (p= 0.025), although only one PDO line was classified as resistant, limiting the strength of patient-level conclusions [12].

#### Short-term models

Prospective validation using short-term models demonstrated strong predictive performance at the individual patient level. In this study, the assay achieved an overall accuracy of 89% (39/44) for predicting first-line platinum-based treatment response, with a sensitivity of 86% (95% CI 72-94) and specificity of 100% (95% CI 64.6-100) when clinical response was assessed using radiologic imaging and/or CA-125 criteria (p < 0.0001) [17].

Additional short-term platform studies using retrospective cohorts reported concordance between PDO sensitivity profiles and RECIST-based or CA-125-based clinical responses. In one study, the RECIST-evaluable subset showed a statistically significant association between in vitro resistance and clinical resistance (p = 0.0037), although the number of evaluable patients was limited, as only 18 patients had RECIST-based clinical response data available for correlation [16]. Another short-term study demonstrated directional concordance between PDO response and clinical outcome but did not report formal statistical testing [20].

#### Long-term models

Prospective evaluation of long-term PDO models also demonstrated high concordance between in vitro PDO drug response and clinical outcomes. In the evaluable cohort (n = 12), PDO-based drug sensitivity testing predicted response to first-line carboplatin/paclitaxel with an accuracy of 91.7%, sensitivity of 100% (95% CI 62.9-100%), and specificity of 66.7% (95% CI 12.53-98.2%), with strong agreement (AUC 0.95; Cohen’s κ = 0.75). In addition, qualitative concordance was observed for PARPi response, where PDO sensitivity or resistance aligned with individual patient outcomes, including cases with discordance between genomic Homologous Recombination Deficiency (HRD) status and observed clinical response [18].

Studies using long-term expandable PDO platforms reported concordance between in vitro drug response and clinical outcome for both standard chemotherapy and targeted agents, primarily in retrospective cohorts [15,19,[21], [22], [23]]. In these studies, concordance was observed at the level of individual patients or small patient subsets rather than through formal predictive accuracy metrics. Notably, one study demonstrated that organoid-clinical correlation depended on culture conditions, with a statistically significant association between PDO response and CA-125 change observed only when organoids were maintained in physiologically relevant medium, highlighting the influence of experimental context on concordance assessment [21].

Taken together, while concordance metrics and statistical analysis varied across studies, alignment between PDO drug response and patient clinical outcome was observed in the majority of included studies. Interpretation of these findings is influenced by differences in study design, clinical endpoint definitions, and analytical approaches, as summarized in Tables 3 and S3.

## Discussion

### Main findings and clinical relevance of PDO-based drug sensitivity testing

This systematic review synthesizes evidence from twelve studies evaluating the ability of PDO models to reflect clinical treatment response in ovarian cancer. Across studies, most analyses reported concordance between in vitro organoid drug sensitivity and patient outcomes, indicating that PDO responses frequently align with clinical sensitivity or resistance patterns. However, only a limited number of studies, primarily prospective validation studies, formally evaluated predictive performance using predefined accuracy metrics at the individual patient level. This distinction is important, as concordance reflects observational agreement between PDO and clinical outcomes, whereas predictive performance requires prospective validation and quantification of the ability of PDOs to predict treatment response. The variability in study design and analytical approaches across the included studies therefore directly influences the strength of the evidence supporting the clinical applicability of PDO-based drug testing.

A key finding of this review is that the most compelling evidence for clinical relevance derives from studies that align experimental design with clinical decision-making. Particularly, prospective or clinically anchored studies using short-term ex vivo platforms demonstrated individual-level predictive performance with defined accuracy metrics (e.g., sensitivity, specificity, and overall accuracy). In contrast, most long-term expandable PDO studies relied on retrospective correlation or subgroup comparisons. These differences do not invalidate either approach, but rather reflect that PDO platforms are currently developed for different translational aims, with short-term models prioritizing rapid prediction of clinical response and long-term platforms emphasizing biological modelling and mechanistic insight. As a result, concordance outcomes must be interpreted within the context of the intended use of each platform.

### Current evidence and clinical validation across cancer types

To place these findings in a broader context, evidence from other tumor types further supports the predictive potential of PDO models, although the level of clinical validation varies. Within gynecologic oncology, evidence beyond ovarian cancer remains limited. In endometrial cancer, PDOs recapitulate the histomorphology and immunohistochemical profile of the original tumor, supporting preservation of tumor identity [26]; however, prospective validation of treatment response prediction at the individual patient level is not yet available. The ongoing PENDOR trial, an observational pilot study, aims to provide proof of concept for their predictive value in endometrial cancer [27]. Similarly, in cervical cancer, PDOs have been established in a cohort of 67 patients and shown to retain key histopathological and genomic features, with functional assays demonstrating interpatient variability in treatment response and concordance with clinical outcomes in selected cases; however, prospective validation of predictive performance remains lacking [28]. Together, these findings indicate that, while PDOs in other gynecologic malignancies demonstrate biological fidelity and early clinical correlations, robust evidence for predictive performance is still emerging. In contrast, stronger evidence has been reported in non-gynecologic cancers. For example, in rectal cancer, PDOs predicted chemoradiation response with 84.43% accuracy, 78.01% sensitivity, and 91.97% specificity [29], whereas in metastatic colorectal cancer, prospective data demonstrated statistically significant correlations between PDO drug sensitivity and radiologic response, with additional associations with survival outcomes [30].

### Limitations affecting clinical translation of PDO models

An important limitation of current ovarian cancer PDO models is that they primarily recapitulate the epithelial compartment of the tumor while lacking important components of the tumor microenvironment, including immune cells, cancer-associated fibroblasts, endothelial cells, and their complex cellular interactions. As a result, although PDOs appear highly promising for predicting responses to cytotoxic chemotherapy and targeted agents acting directly on tumor cells, their ability to predict responses to immunotherapies or therapies that rely on stromal or immune interactions remains limited. Future developments, including co-culture systems and organoid-on-chip platforms that incorporate immune and stromal components, may provide more physiologically relevant models and further improve the clinical predictive value of PDOs as precision oncology tools.

Another important limitation in current PDO drug sensitivity studies is the extrapolation of single-agent in vitro responses to clinical outcomes following combination chemotherapy. Several included studies [12,19] evaluated sensitivity to individual drugs, whereas ovarian cancer treatment commonly consists of combination regimens. Although single-agent testing can provide insights into intrinsic tumor sensitivity, it may not fully capture drug interactions, synergistic effects, or pharmacokinetic differences occurring in patients. Therefore, single-agent assays may result in false-positive or false-negative predictions when used to estimate clinical efficacy of combination treatments. In addition, variability in drug exposure conditions represents an important challenge for clinical translation. The recent consensus on PDO-based drug sensitivity testing highlights that in vitro drug concentrations may not directly reflect clinical exposure and recommends greater standardization of drug concentration selection, including approaches based on dose-response curves or clinically relevant concentrations such as maximum plasma concentration, to improve reproducibility and comparability between studies [31].

### Methodological challenges and sources of bias in PDO drug sensitivity testing

#### Methodological heterogeneity

Methodological heterogeneity across studies represents a central challenge for data synthesis. Variability in organoid platforms, drug exposure strategies, response readouts, and clinical endpoint definitions limit direct comparability of concordance metrics and complicate generalization. Importantly, discordance between in vitro testing conditions and clinical treatment regimens, such as testing single agents when patients received combination therapy, may systematically weaken or mask true predictive relationships, as the effects of combination regimens cannot always be captured by single-agent testing. Similarly, the use of multiple clinical endpoints without a predefined hierarchy increases the risk of selective reporting, particularly in small cohorts.

#### Risk of bias and limited prospective validation

The strongest evidence for the clinical relevance of PDO models comes from two prospective validation studies, which demonstrated individual-level predictive performance using predefined accuracy metrics, with reported accuracies of 89% and 91.7% [17,18]. However, the majority of included studies were retrospective or feasibility-focused, which inherently increases susceptibility to confirmation bias and limits the strength of causal inference. Furthermore, several sources of bias may impact the robustness of the available evidence. Selection bias is inherent to PDO studies, as inclusion depends on sufficient viable tissue and successful organoid establishment, potentially enriching for biologically favorable tumors. Attrition bias may arise from the exclusion of failed cultures or non-evaluable clinical outcomes, which are not consistently reported across studies. Although most studies assessed tumor-PDO comparability, the depth and methodology of validation varied substantially, influencing confidence that measured drug responses accurately reflect the originating tumor biology. Together with heterogeneity in study design, clinical endpoints, and analytical approaches, these limitations made it impossible to perform a quantitative meta-analysis.

#### Future perspectives

Despite these limitations, a notable strength of the current literature is the consistency of directional concordance across heterogeneous platforms, suggesting that PDOs capture clinically meaningful aspects of tumor drug response. Rather than undermining the field, the observed heterogeneity reflects an early stage of clinical translation in which platforms are being developed for different clinical and research objectives. Moving forward, the key challenge is not to eliminate methodological diversity, but to establish standardized guidelines for clinical endpoint selection, alignment between in vitro and clinical drug testing, and validation criteria, thereby enabling better cross-study comparison. Future prospective studies should therefore use clinically matched treatment regimens, including combination therapies where applicable, predefined endpoints such as RECIST response and progression-free survival, and standardized measures of predictive performance, including sensitivity, specificity, and accuracy. Consistent reporting of organoid establishment rates and culture failures, together with multicenter validation in larger patient cohorts, will be essential to determine the clinical utility of PDO-guided treatment prediction in ovarian cancer.

## Conclusions

This systematic review of twelve studies evaluating PDO models in ovarian cancer demonstrates that, across diverse experimental platforms and clinical contexts, organoid drug responses were frequently concordant with patient treatment outcomes. Both short-term ex vivo models and long-term expandable PDO platforms showed the ability to reflect clinical sensitivity or resistance to chemotherapy and targeted agents, although the strength and statistical support of these associations varied between studies. Despite methodological heterogeneity and limited cohort sizes, the available evidence indicates a consistent trend toward the potential use of PDOs as functional models for predicting therapeutic response in ovarian cancer. Further prospective studies using standardized clinical endpoints and drug testing methodologies are now needed to validate and define the clinical applicability of PDOs.

## Funding

This research did not receive any specific grant from funding agencies in the public, commercial, or not-for-profit sectors.

## CRediT authorship contribution statement

**Merel Vegter:** Writing – review & editing, Writing – original draft, Visualization, Project administration, Methodology, Investigation, Formal analysis, Data curation, Conceptualization. **Prakriti Garkhail:** Writing – review & editing, Writing – original draft, Methodology, Investigation, Data curation. **Gatske M. Nieuwenhuyzen-de Boer:** Writing – review & editing, Supervision. **Ingrid A. Boere:** Writing – review & editing, Supervision. **Heleen J. van Beekhuizen:** Writing – review & editing, Funding acquisition.

## Declaration of competing interest

The authors declare that they have no known competing financial interests or personal relationships that could have appeared to influence the work reported in this paper.

## Appendix Supplementary materials

Image, application 1
