# Supplementary material for: Patient-derived organoids as predictor of clinical response in ovarian cancer: a systematic review
Source: Transl Oncol. 2026 Sep 6;73:103017. doi: 10.1016/j.tranon.2026.103017 (PMC13572739; doi:10.1016/j.tranon.2026.103017)
Supplement: Supplementary file 1 [file mmc1.docx]

# Supplementary materials

## Table S1. Full search strategies for each database

| **Database** | **Records retrieved** | **Full search strategy** |
| --- | --- | --- |
| Embase | 471 | (organoid/de OR (organoid*):ab,ti,kw) AND (‘ovary tumor’/exp OR (((ovar*) NEAR/3 (cancer* OR carcinoma* OR tumor* OR tumour* OR malignan* OR neoplas*))):ab,ti,kw) AND (‘treatment response’/exp OR ‘personalized medicine’/exp OR ‘preclinical model’/de OR ‘angiogenesis inhibitor’/exp OR (respons* OR personali* OR preclinical OR angiogenes*):ab,ti,kw) NOT (([Conference Abstract]/lim OR [Conference Review]/lim) AND [1800–2020]/py) AND [ENGLISH]/lim |
| MEDLINE (PubMed) | 187 | (Organoids/ OR (organoid*).ab,ti,kf.) AND (exp Ovarian Neoplasms/ OR (((ovar*) ADJ3 (cancer* OR carcinoma* OR tumor* OR tumour* OR malignan* OR neoplas*))).ab,ti,kf.) AND (exp Precision Medicine/ OR exp Angiogenesis Inhibitors/ OR (respons* OR personali* OR preclinical OR angiogenes*).ab,ti,kf.) NOT ((congres* OR abstract*).pt. AND 1800:2020.(sa_year).) AND (english).la. |
| Cochrane Library | 0 | ((organoid*):ab,ti,kw) AND ((((ovar*) NEAR/3 (cancer* OR carcinoma* OR tumor* OR tumour* OR malignan* OR neoplas*))):ab,ti,kw) AND ((respons* OR personali* OR preclinical OR angiogenes*):ab,ti,kw) NOT ("conference abstract":kw OR Trial registry record:pt) |
| Web of Science | 224 | TS=(((organoid*)) AND ((((ovar*) NEAR/2 (cancer* OR carcinoma* OR tumor* OR tumour* OR malignan* OR neoplas*)))) AND ((respons* OR personali* OR preclinical OR angiogenes*))) NOT DT=(Meeting Abstract OR Meeting Summary) AND LA=(English) |
| Google Scholar | 50 | organoid \| organoids “ovary\|ovarian\|ovarium cancer\|carcinoma\|tumor\|tumour\|malignancy\|neoplasm” “treatment response”\|personalized\|personalised\|“preclinical model”\|angiogenesis |

## Table S2. PDO derivation, culture, validation, and drug testing methodology by study.

| **Study** | **Design** | **N patients / N PDO lines** | **Tumor tissue source and site** | **Ovarian cancer subtype(s)** | **3D model type + culture format (incl. matrix)** | **Medium composition (as reported)** | **Time to drug testing readout** | **Establishment outcome (attempted/success/failed)** | **Fidelity / tumor–PDO comparability assessment** | **Drug sensitivity assay/readout** | **Drugs tested** | **Concentration strategy / thresholds** |
| --- | --- | --- | --- | --- | --- | --- | --- | --- | --- | --- | --- | --- |
| **de Witte 2020** [11] | Retrospective translational correlation | 23 / 36 | Tumor tissue and ascites-derived samples (both used across cohort) | Mixed EOC histologies (multiple) | BME-embedded long-term PDOs (Cultrex BME type 2) | Advanced DMEM/F12 + GlutaMAX/HEPES/antibiotics; WNT/R-spondin/Noggin conditioned media; B27; nicotinamide; N-acetylcysteine; hydrocortisone; heregulin β-1; FGF10; EGF; forskolin; A83-01; Y-27632; β-estradiol | Pilot “rapid” feasibility reported within ~20 days (subset) | Not reported as attempts vs failures (biobank panel analyzed as established lines) | Histology + genomic characterization supporting retention of tumor features | Dose–response; IC50/AUC; interpretation vs achievable plasma exposure | Alpelisib, adavosertib, afatinib, AZD8055, carboplatin, gemcitabine, MK-2206, niraparib, olaparib, paclitaxel, pictilisib, rucaparib, vemurafenib, flavopiridol | High/low response interpreted relative to clinically achievable Css/Cmax benchmarks |
| **Gorski 2021** [12] | Retrospective cohort | 6 / 6 | Surgical tissue (primary or interval debulking) | HGSC only | Long-term PDO lines (matrix-based organoid culture) | Culture uses published organoid conditions | Not reported | 6 established lines (attempt denominator not explicitly stated beyond cohort) | Mechanistic molecular profiling performed; formal paired tumor–PDO fidelity testing not comprehensively reported | Viability curves; EC50 | Carboplatin | Carboplatin 0.1–100 µM; resistance if EC50 exceeds clinically achievable exposure |
| **Shuford 2019** [17] | Prospective, blinded validation + feasibility | 44 / 44 (validation cohort); 83 successful assays in feasibility set | Fresh tumor tissue from primary debulking or laparoscopic biopsy | High-grade epithelial OC/FT/PP cancers (newly diagnosed cohort) | Ex vivo 3D spheroids (384-well spheroid plates); no long-term expansion | Not reported (rapid ex vivo spheroid assay) | ~7 days from tissue receipt | Feasibility: 83 successful assays; validation performed in 44 newly diagnosed evaluable patients | Assay QC and epithelial tumor confirmation (no paired genomic matching reported) | CellTiter-Glo 3D; IC50-based classification; ROC-derived cutoffs (carboplatin) | Carboplatin, paclitaxel, doxorubicin, gemcitabine, topotecan, olaparib | Drug dosing 0.005–100 µM; 72h exposure; carboplatin cutoffs via ROC |
| **Senkowski 2023** [21] | Platform development + retrospective correlation | 10 / 17 long-term cultures (from 19 attempted patients) | Tumor tissue, biopsies, and ascites (multiple sites; translated to plain language) | HGSC only | BME-embedded PDOs; parallel expansion in M1 and M2; drug profiling also in HPLM | Basal Advanced DMEM/F12 + Primocin + HEPES + NAC + GlutaMAX + B27; M1 = M0.3 + nicotinamide; M2 = M1 + EGF + heregulin β-1 + hydrocortisone + forskolin; testing also in HPLM | Drugs added 7–10 days post-seeding; 96h exposure | Attempted 19 → successful long-term cultures in 10 patients (53%); 17 stable cultures | WGS/IHC/scRNA-seq show organoids retain genetic/phenotypic features of original tumor; success required retention of sample TP53 mutation | Imaging viability (Hoechst/CellTox); AUC; media-dependent effects assessed | Carboplatin, paclitaxel, gemcitabine, olaparib, niraparib, rucaparib, adavosertib | Carboplatin 10 nM–100 µM; others 1 nM–10 µM; includes carboplatin + paclitaxel combination |
| **Ito 2023** [16] | Cohort with RECIST-linked subset | 61 / 61 primary organoids; TC-treated subset 50; assay success 42 | Surgical specimens and ascites (Cancer tissue-originated spheroids method) | Serous, endometrioid, clear cell, mucinous (discussed) | Cancer Tissue-Originated Spheroids suspension spheroids; poly-HEMA plates (no matrix dome) | StemPro hESC medium (suspension Cancer tissue-originated spheroids culture) | 7 days drug exposure (primary assay) | Organoid prep success 61/61; drug assay success 42/50 (84%) | Histology + IHC reported as consistent with original tumors | ATPlite 1step ATP viability day 7; IC50 by Prism; normalized to spheroid area | Paclitaxel, carboplatin (clinical prediction); additional screening performed on expanded lines | Paclitaxel 0–1000 nM; carboplatin 0–1000 µM; 7-day exposure |
| **Tao 2022** [19] | Retrospective translational correlation (small cohort) | 7 / 7 (chemo-naïve correlation cohort) | Tumor tissue (chemo-naïve cohort described) | EOC including HGSC (multiple) | Long-term PDO culture (matrix-embedded) | Complete medium per Kopper et al. ovarian cancer PDO protocol | Drug added day 4; viability after 5 days treatment | Not reported as attempt denominator; cohort analyzed as established PDOs | Paired genomic concordance described; HRR-gene PVs and mutational signatures assessed in tumors/PDOs | CellTiter-Lumi; IC50 via GraphPad | Cisplatin (clinical correlation); PARPi response explored in study | Response interpretation includes comparison to Css/Cmax benchmarks; cisplatin IC50 compared to Cmax |
| **Jun 2023** [20] | Platform/high-throughput + clinical linkage | 10 / 10 | Surgical tumor specimens | HGSC; low-grade serous; endometrioid; clear cell; carcinosarcoma | Organo-on-pillar (High-TOP) micropillar platform; 50% Matrigel microdrops | Ovarian cancer organoid medium” referenced; full recipe provided in article | Stabilization then 5–7 days drug exposure | Not reported as attempts vs failures (cohort presented as successfully profiled PDOs) | Histology/IHC comparison shown (tumor markers) | Calcein imaging ± CellTiter-Glo 3D; normalized AUC | Carboplatin (clinical linkage set) | 6 doses; max 250 µM; dilution factor 3 |
| **Chang 2024** [15] | Feasibility/biobank + drug testing | Attempts 13; successes 7 (54%); drug testing on established PDOs | Tumor tissue including metastatic site in at least one HGSC (omentum noted) | HGSC; mucinous; clear cell; carcinosarcoma | Matrigel dome PDOs (70% Matrigel / 30% DMEM/F12; dome seeding described) | DMEM/F12 base with B27, NAC, WNT3A, R-spondin1, Noggin, nicotinamide, EGF, FGF10, forskolin, A83-01, hydrocortisone, heregulin β-1, β-estradiol, Y-27632 (as described) | Organoids typically formed within ~1–3 weeks before testing | 7/13 establishment successes (54%) | Histology/IHC concordance; sequencing/CNV comparisons reported between tumor and PDO | CellTiter-Glo 3D; viability-based sensitivity | Carboplatin, paclitaxel, gemcitabine, doxorubicin, olaparib | Concentrations based on published IC50 references; serial dilution (details in paper) |
| **Chen 2024** [10] | Feasibility + clinical correlation (including 4 clinical cases) | 31 PDOs established; 28 expandable; 15 ovarian PDOs; 10 ovarian PDOs drug-tested | Surgical resections (case narratives include interval debulking in an example) | HGSC, clear cell, mucinous, endometrioid/dedifferentiated endometrioid (reported) | Matrix dome PDOs | Advanced DMEM/F12 + HEPES + GlutaMAX + B27 + N2 + EGF + FGF10 + 5% Wnt3A conditioned medium + R-spondin + Noggin + pen/strep + Y-27632 + nicotinamide + SB431542; passaging 1:2–3 every 1–4 weeks | Not reported | Establishment: 31; expandable: 28 | Histology + IHC compared to parental tumors (TP53, PAX8, WT1, CK7, CK20, Ki-67) | CellTiter-Glo 3D; AUC used for drug comparisons | Cisplatin, carboplatin, paclitaxel, gemcitabine, epirubicin, doxorubicin, topotecan, olaparib | Dose–response curves; AUC reported; exact concentration series not specified in methods text |
| **Nikeghbal 2025** [22] | Retrospective translational feasibility study | 5/5 | Malignant ascites-derived cancer spheroids | HGSC | Matrix-embedded organoids (Ultimatrix domes; 3D culture) | Advanced DMEM/F12 + GlutaMAX + HEPES + Pen/Strep + Noggin + R-spondin-1 + FGF10 + heregulin β-1 + EGF + B27 + N-acetylcysteine + β-estradiol + nicotinamide + A83-01 + Y-27632 + forskolin + hydrocortisone | Organoid formation ~6–14 days; drug exposure 72h | 5 PDO models established (no explicit failure rate reported; all expanded ≥9 passages) | Only phenotypic (function response based) validation, e.g. no genomic/ICH comparison performed | CellTiter-Glo 3D viability assay | Carboplatin, paclitaxel, carboplatin + paclitaxel | Fixed treatment concentrations (as per figures); 72h exposure; no IC50-based thresholding reported |
| **Thorel 2025** [23] | Retrospective translational correlation study | 31 / 37 long-term PDO models | Surgical resections, including both primary tumors and metastatic sites and ascites | Multiple EOC subtypes (HGSC, LGSOC, endometrioid, mucinous, clear cell) | BME2-embedded long-term PDO cultures (matrix dome) | Advanced DMEM + pen/strep + GlutaMAX + B27 + N-acetylcysteine + EGF + FGF10 + FGF-basic + A83-01 + Y-27632 + SB202190 + nicotinamide + PGE2 + R-spondin-1 conditioned medium + L-WRN conditioned medium | Organoids treated when reaching ~75–150 µm; drug exposure ~7 days | 31 patients → 37 long-term PDO models (success rate not explicitly quantified) | Histology, IHC, copy number variation, and transcriptomic profiling demonstrate strong tumor–PDO concordance | CellTiter-Glo 3D (ATP-based viability); AUC and normalized z-scores | Carboplatin, paclitaxel, gemcitabine, doxorubicin, olaparib, niraparib | Multi-dose dose–response curves; AUC and z-score–based relative sensitivity (no fixed clinical IC50/Cmax thresholds) |
| **Wang 2026** [18] | Prospective clinical validation study | 30 patients → 21 PDO lines | Primary tumor tissue from surgery or biopsy (treatment-naïve patients) | HGSC (majority), LGSOC, endometrioid, mucinous (minority) | Matrigel-embedded PDOs (GFR Matrigel dome culture) | EOC organoid medium based on Kopper et al. protocol (modified; full composition in supplementary data) | Organoids formed within ~7–10 days; drug exposure 6 days | 30 patients → 21 successful PDO lines (70%) | Histology (H&E), IHC, and whole-exome sequencing confirm preservation of tumor features | CellTiter-Glo 3D; IC50-based dose–response curves | Carboplatin, paclitaxel, olaparib, niraparib | 6-dose series per drug; IC50 calculated from dose–response curves |

Abbreviations: A83-01 = TGF-β receptor inhibitor; AUC = Area Under the Curve; ATP = Adenosine Triphosphate; ATPlite = ATP-based luminescence viability assay; B27 = Serum-free supplement for cell culture; BME = Basement Membrane Extract; BME2 = Basement Membrane Extract type 2; CK7 = Cytokeratin 7; CK20 = Cytokeratin 20; CNV = Copy Number Variation; Css = Steady-State Concentration; Cmax = Maximum Plasma Concentration; DMEM/F12 = Dulbecco’s Modified Eagle Medium/Nutrient Mixture F-12; EC50 = Half Maximal Effective Concentration; EGF = Epidermal Growth Factor; EOC = Epithelial Ovarian Cancer; FGF10 = Fibroblast Growth Factor 10; GFR = Growth Factor Reduced; GlutaMAX = Stabilized L-glutamine supplement; H&E = Hematoxylin and Eosin staining; HEPES = 4-(2-hydroxyethyl)-1-piperazineethanesulfonic acid buffer; HGSC = High-Grade Serous Carcinoma; HPLM = Human Plasma-Like Medium; HRR = Homologous Recombination Repair; IHC = Immunohistochemistry; IC50 = Half Maximal Inhibitory Concentration; LGSOC = Low-Grade Serous Ovarian Carcinoma; L-WRN = Conditioned medium containing Wnt3A, R-spondin, and Noggin; M1 = Organoid medium condition 1 (baseline + nicotinamide); M2 = Organoid medium condition 2 (M1 + additional growth factors); Matrigel = Basement membrane matrix derived from Engelbreth-Holm-Swarm tumor; NAC = N-acetylcysteine; N2 = Serum-free supplement for neural cell culture; PARPi = Poly(ADP-ribose) Polymerase inhibitor; PDO = Patient-Derived Organoid; PGE2 = Prostaglandin E2; poly-HEMA = Poly(2-hydroxyethyl methacrylate); Primocin = Broad-spectrum antibiotic for cell culture; scRNA-seq = Single-cell RNA sequencing; SB202190 = p38 MAP kinase inhibitor; SB431542 = TGF-β receptor inhibitor; TP53 = Tumor Protein p53 gene; WT1 = Wilms Tumor 1 protein; WGS = Whole Genome Sequencing; Wnt3A = Wnt family member 3A growth factor; Y-27632 = Rho-associated protein kinase (ROCK) inhibitor; β-estradiol = Estrogen hormone (estradiol); heregulin β-1 = Neuregulin 1 growth factor variant.

## Table S3. Clinical endpoints used to define therapeutic response and concordance/association between PDO response and patient outcomes.

| **Study** | **Clinical endpoints used to define therapeutic response** | **Clinical therapy received by patient** | **In vitro PDO drug(s) tested for comparison** | **Organoid response metric** | **Concordance / association results (as reported)** | **Clinical regimen and in vitro matching** |
| --- | --- | --- | --- | --- | --- | --- |
| **de Witte 2020** [11] | CRS (histopathology), CA-125 normalization, radiologic response (RECIST based) SD vs PR; PFS/OS strata | Standard-of-care systemic therapy (varies across cohort) | Multiple agents; includes platinum-taxane and targeted drugs | IC50/AUC; response interpreted vs Css/Cmax | PDO combination chemo response differed significantly by: CRS1 vs CRS2 (p=5.821e−05); CA-125 normalization vs not (p=0.0004); RECIST SD vs PR (p=0.0092) | Some combinations tested (e.g., carboplatin + paclitaxel) alongside single agents |
| **Gorski 2021** [12] | PFS (RECIST-defined recurrence/progression timing) | Platinum-based chemotherapy ± bevacizumab ± PARPi | Carboplatin | EC50 | One PDO classified resistant (EC50 > achievable exposure) associated with shorter PFS (p=0.025); EC50 differed vs sensitive group (p=0.018) | Clinical regimens typically combination; in vitro comparison single-agent carboplatin |
| **Shuford 2019** [17] | CA-125 + radiographic assessment (adjuvant); RECIST 1.1 (neoadjuvant) | First-line platinum-based therapy (predominantly carboplatin + paclitaxel) | Single agents: carboplatin, paclitaxel, doxorubicin, gemcitabine, topotecan, olaparib | IC50-based classifier; ROC-based carboplatin cutoff | Prospective validation cohort (44): accuracy 89% (39/44); sensitivity 86% (95% CI 72–94); specificity 100% (95% CI 64.6–100); PFS stratification reported | Clinical regimen combination; in vitro single-agent testing; classified as responder if sensitive to ≥1 administered agent |
| **Senkowski 2023** [21] | CA-125 change attributable to carboplatin + paclitaxel (matched sampling) | Carboplatin + paclitaxel | Carboplatin + paclitaxel (and components) | AUC | Correlation depends on medium: expansion media r=0.552, p=0.105 (NS); HPLM r=0.770, p=0.013 (significant) | Clinical regimen combination; in vitro tested combination and components |
| **Ito 2023** [16] | RECIST 1.1 (measurable disease); CA-125 criteria for non-measurable; imaging by CT or MRI | Paclitaxel + carboplatin | Paclitaxel and carboplatin | IC50 ranking; “double-sensitive” / “double-resistant” | RECIST-evaluable subset showed significant association: clinical resistant cases aligned with in vitro resistance patterns; p=0.0037 (Fisher’s exact) | Clinical combination; in vitro single-agent testing for both components then combined classification |
| **Tao 2022** [19] | CA-125 normalization (GCIG); RECIST 1.1 by CT; recurrence timing/platinum sensitivity definition (>6 months) | Platinum–taxane, PARPi (subset of patients) | Cisplatin (for clinical correlation analysis) | IC50; comparison to Cmax | PDO cisplatin response aligned with CA-125 normalization (p=0.0047). RECIST responses (PR/CR in all) did not correlate. Early recurrence at 2 months (platinum-resistant) aligned with least cisplatin-responsive PDO | Clinical combination; in vitro single-agent cisplatin used as platinum proxy |
| **Jun 2023** [20] | Platinum sensitivity status (refractory vs sensitive), supported by radiologic response (CT/PET-CT) and CA-125 trends | Predominantly platinum-taxane ± bevacizumab | Carboplatin | Normalized AUC | Patient-linked feasibility/correlation presented; no single cohort-level accuracy/sensitivity metric reported across all 10 | Clinical combination; in vitro single-agent carboplatin in platform linkage |
| **Chang 2024** [15] | Follow-up/outcomes reported descriptively in case table | Paclitaxel + carboplatin ± bevacizumab | Carboplatin, paclitaxel, gemcitabine, doxorubicin, olaparib | Viability-based sensitivity (CellTiter-Glo 3D) | Case-linked in vitro sensitivities reported alongside clinical course; no single consolidated concordance statistic reported | Clinical combination; in vitro single-agent panel |
| **Chen 2024** [10] | Platinum sensitivity status (sensitive vs resistant vs refractory), based on clinical course (treatment response, recurrence/progression, imaging, and CA-125 trends) | Regimens varied (Paclitaxel, carboplatin, gemcitabine, bevacizumab and liposomal doxorubicin) | Cisplatin, carboplatin, paclitaxel, gemcitabine, epirubicin, doxorubicin, topotecan, olaparib | AUC / dose–response | Four case correlations reported: PDO drug response patterns “correlated well” with platinum-sensitive vs resistant vs refractory clinical courses; no cohort-level accuracy metric reported | Clinical combinations; in vitro single-agent testing |
| **Nikeghbal 2025** [22] | Platinum sensitivity classification based on platinum-free interval  Clinical response to platinum-based chemotherapy ((platinum-sensitive ≥6 months, resistant <6 months, refractory no response). And they used; descriptive clinical outcome. | First-line platinum-based chemotherapy (carboplatin + paclitaxel) | Carboplatin, paclitaxel, and combination | Viability assay (CellTiter-Glo 3D) | PDO responses reflected clinical platinum sensitivity categories, with platinum-sensitive cases showing marked viability reduction and resistant/refractory cases showing minimal response; no quantitative concordance metrics reported | Clinical combination; in vitro both single agents and combination tested |
| **Thorel 2025** [23] | Clinical response to first-line platinum-based chemotherapy (platinum sensitivity context). Treatment response classification (sensitive vs resistant) with PFS. | Standard-of-care platinum–taxane chemotherapy ± targeted therapy (PARPi in subset) | Carboplatin, paclitaxel, doxorubicin, gemcitabine, PARPi (olaparib, niraparib) | AUC (normalized AUC z-scores from viability assays) | PDO carboplatin response was associated with patient platinum-free interval, distinguishing resistant vs sensitive cases, though not statistically significant (p=0.06). | Clinical combination; in vitro multi-drug panel including both single agents and targeted therapies |
| **Wang 2026** [18] | RECIST-based response to first-line chemotherapy; PARPi response during maintenance and PFS | Carboplatin + paclitaxel (first-line) followed by PARPi maintenance (subset) | Carboplatin, paclitaxel, olaparib, niraparib | IC50; ROC/AUC-based prediction model | Organoid drug sensitivity testing accurately predicted clinical response to first-line chemotherapy (accuracy 91.7%, sensitivity 100%, specificity 66.7%; AUC 0.95) and correctly identified PARPi response, including cases discordant with genomic HRD status. | Clinical combination; in vitro testing included both combination-relevant agents and PARPi |

Abbreviations: AUC = Area Under the Curve; CA-125 = Cancer Antigen 125; CI = Confidence Interval; CR = Complete Response; CRS = Chemotherapy Response Score; CT = Computed Tomography; EC50 = Half Maximal Effective Concentration; GCIG = Gynecologic Cancer InterGroup; HPLM = Human Plasma-Like Medium; HRD = Homologous Recombination Deficiency; IC50 = Half Maximal Inhibitory Concentration; MRI = Magnetic Resonance Imaging; NS = Not Significant; OS = Overall Survival; PARPi = Poly(ADP-ribose) Polymerase inhibitor; PFS = Progression-Free Survival; PDO = Patient-Derived Organoid; PR = Partial Response; RECIST = Response Evaluation Criteria in Solid Tumors; ROC = Receiver Operating Characteristic; SD = Stable Disease
